# Ophthalmic Drug Delivery Systems for Antibiotherapy—A Review

**DOI:** 10.3390/pharmaceutics10010010

**Published:** 2018-01-13

**Authors:** Marion Dubald, Sandrine Bourgeois, Véronique Andrieu, Hatem Fessi

**Affiliations:** 1 Univ Lyon, Université Claude Bernard Lyon 1, Centre National de la Recherche Scientifique (CNRS), Laboratoire d’Automatique et de GEnie des Procédés (LAGEP) Unité Mixte de Recherche UMR 5007, 43 boulevard du 11 novembre 1918, F-69100, Villeurbanne, France; Marion.Dubald@horus-pharma.fr (M.D.); sandrine.bourgeois@univ-lyon1.fr (S.B.); 2 Horus Pharma, Cap Var, 148 avenue Georges Guynemer, F-06700 Saint Laurent du Var, France; 3 Univ Lyon, Université Claude Bernard Lyon 1, Institut des Sciences Pharmaceutiques et Biologiques (ISPB)—Faculté de Pharmacie de Lyon, 8 avenue Rockefeller, F-69008, Lyon, France; 4 Unité de Recherche sur les Maladies Infectieuses et Tropicales Émergentes (URMITE), Unité Mixte de Recherche 6236 Centre National de la Recherche Scientifique (CNRS), Aix Marseille Université, Faculté de Médecine et de Pharmacie, F-13005 Marseille, France; veronique.andrieu@univ-amu.fr

**Keywords:** antibiotics, ocular drug administration, nanoparticles, drug delivery

## Abstract

The last fifty years, ophthalmic drug delivery research has made much progress, challenging scientists about the advantages and limitations of this drug delivery approach. Topical eye drops are the most commonly used formulation in ocular drug delivery. Despite the good tolerance for patients, this topical administration is only focus on the anterior ocular diseases and had a high precorneal loss of drugs due to the tears production and ocular barriers. Antibiotics are popularly used in solution or in ointment for the ophthalmic route. However, their local bioavailability needs to be improved in order to decrease the frequency of administrations and the side effects and to increase their therapeutic efficiency. For this purpose, sustained release forms for ophthalmic delivery of antibiotics were developed. This review briefly describes the ocular administration with the ocular barriers and the currently topical forms. It focuses on experimental results to bypass the limitations of ocular antibiotic delivery with new ocular technology as colloidal and in situ gelling systems or with the improvement of existing forms as implants and contact lenses. Nanotechnology is presently a promising drug delivery way to provide protection of antibiotics and improve pathway through ocular barriers and deliver drugs to specific target sites.

## 1. Introduction

Ophthalmic drug delivery presents major challenges for pharmaceutical and medicinal sciences. For several decades, progress has been achieved to improve the currently dosage forms. Ocular diseases are complicated to treat, and ocular forms need to be safe, non-allergic for the patient and sterile. Topical forms represent 90% of the marked formulation [1]. The tear fluid turnover, the nasolacrimal drainage, the corneal epithelium and the blood-ocular barriers are decreasing the local bioavailability of drugs and residence time on the ocular surface in topical application. Only 5%–10% of the drug crosses the corneal barriers. Anterior segment diseases as blepharitis, conjunctivitis, scleritis, keratitis and dry eye syndrome are resolved with topical or periocular administration. The delivery of drug to the posterior segment of the eye for glaucoma, endophthalmitis or uveitis and to the anterior segment has the same issue of poor bioavailability of the drug and barriers. However, intraocular administration might be preferred despite its risk of complication [2]. In addition, compared to the oral route, ocular drug delivery provided equivalent or better bioavailability in the eye [3]. Approaches have been made for the improvement of the bioavailability of the drug, the controlled release and the improvement of the therapeutic effect [4].

Antibiotics are group of medicines popularly used in ophthalmic delivery due to the multiples ocular diseases (microbial keratitis, conjunctivitis, Meibomian gland dysfunction and dry eye). Infectious disease is one of the most public health challenge [5]. Antibacterial therapies can be administrated in the eye by topical, subtenon, intraocular or subconjunctival administration. Tetracyclines, fluoroquinolones, aminoglycosides and penicillins are examples of antibiotics commonly used in the treatment of eye infections [6]. The antimicrobial resistance is the ability of bacteria to resist to the effect of an antibiotic administration. This limitation of efficacy is caused by the misuse of antibiotic, the overuse of this group of medicine and the adaptation of the bacteria to the effect. In fact, ophthalmic antibiotic delivery aims to decrease the frequency of administration and dosing by improving the current forms and developing new ones.

New ocular drug delivery forms are various; they included in situ gelling systems, liposomes, nanoparticles, niosomes, nanoemulsions and microemulsions. They are suitable for hydrophilic or lipophilic drugs, have the capacity of targeting a specific site and can be administrated in different routes. With the appropriate excipients, in situ gelling systems are able to increase the precorneal residence time and decrease the loss of drug due to the tear. Different polymers, methods of preparation and compositions allow the nanoparticles to respond to a need for mucoadhesion, topical, periocular or intraocular administration, and to obtain a stable, effective and non-irritating formulation for the patient.

The objective of this paper is to review the antibiotic formulations for an ophthalmic administration. First the ocular anatomy and physiology and the ocular barriers were described. Topical forms such as eye drops, ointments, hydrogels, contact lenses and ophthalmic inserts are developed in a second part to introduce the ocular administration and explain the currently marketed dosage form. Finally, recent advances on ocular antibiotic administration are reviewed. In vitro and in vivo studies explored the efficacy of antimicrobial formulations. Different compositions and forms are developed to improve the bioavailability of antibiotics, increase the residence time in the eye and the therapeutic response.

## 2. Anatomy and Physiology of the Eye for Ocular Drug Delivery

### 2.1. Anatomy and Physiology of the Eye

The eye has a spherical shape included in the orbital cavity and protected by lids. With a diameter of 24 mm and a volume of 6.5 cm^3^, it weighs about 7.5 g.

**Figure 1:**
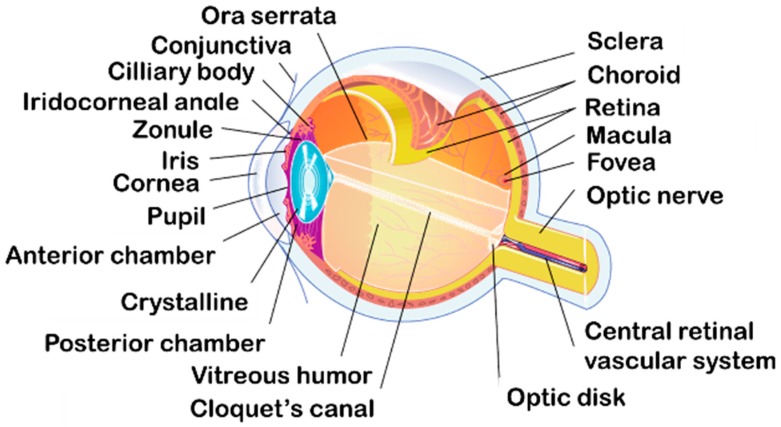
Schematic illustration of ocular structures and barriers.

Several layers with specifics structures compose the eyeball and divide it in two segments [3,7]: the anterior segment (cornea, conjunctiva, aqueous humor, iris, ciliary body and lens) and the posterior segment (retina, choroid, sclera and vitreous humor) as illustrated in Figure 1.

#### 2.1.1. Three Different Layers

The eye is surrounded by three different layers: the outer layer, the medium layer and the inner layer. The outer layer is composed by the cornea and the sclera. They are fibrous tissue and have a protective function for the eyeball. The sclera, continuous with the cornea, is an avascular, white, strong, and elastic tissue. It covers 80% of the eye’s tunic. The cornea, joining the sclera at the limbus, is a thin (0.5 mm) [8], avascular and transparent layer which allows the light penetration to the globe. The anterior and posterior segments of the eye are anatomically separated by the sclera and the cornea (Figure 1).

The middle layer is a vascular envelope also called uvea, formed by the iris, the choroid and the ciliary body. The iris is a contractile, circular membrane opened at its center by the pupil. It is the color part of the eye located to the posterior region of the cornea. At the posterior of the uvea, the choroid is a highly vascularized membrane. It supplies nutriments and oxygen to the iris and retinal photoreceptors. Between the sclera and the retina, the ciliary body secrets the aqueous humor with the ciliary processes and contains smooth muscles that control the shape of the lens.

The innermost tissue is the retina. It is the neuronal tissue responsible of the vision composed of two types of tissues. The retina as the choroid, cover the inside of the posterior segment from the optic nerve to the *ora serrata*. The neural tissue is composed by the photoreceptor (rods for the night and the peripheral vision and cones for the color and the details), the bipolar cells and the ganglion cells.

#### 2.1.2. Inside the Globe

The inside of the eye is composed of three major compounds: the crystalline, the aqueous humor and the vitreous humor.

The crystalline is a biconvex, transparent lens located behind the iris and the pupil. It is an avascular, elastic organ connected to the optical layer by the ciliary body. The crystalline separates the aqueous humor from the vitreous humor. Its function is to allow the accommodation by concentrating the light on the retina with its contraction.

The aqueous humor is a clear optical fluid with low viscosity. Located in the anterior and the posterior chambers of the eye, the aqueous humor is continuously formed by the ciliary body (2.4 ± 0.6 µL/min in humans) [9]. The anterior chamber and the posterior chamber contain 0.250 mL and 0.060 mL of aqueous humor respectively. Composed by 99% of water the aqueous humor supplies nutriments to the iris, the crystalline and the cornea [10]. It also maintains the intraocular pressure of the eye and the convex form of the lens.

The vitreous body, also called vitreous humor, is located between the crystalline and the retina. It is a transparent and gelatinous liquid, which represents 90% of the volume of the eye (4.0 mL). Composed of 99% of water, it helps to maintain the structure of the eyeball and plays the role of a lens in the delivery of the light ray.

#### 2.1.3. Ocular Annexes

Ocular annexes represent the external anatomic parts of the eye necessary for the proper functioning of the ocular apparatus as the muscles, the eyelids and the lacrimal apparatus.

The six extraocular muscles induce the movement of the eye in the orbit and the control of the superior eyelid movement. The eyelids are the first protection for the eye. They are movable folds of skin that covers the ocular surface, hydrate the cornea and clean the surface of the eye from debris. The superior eyelid regulates the light reaching the eye using extraocular muscles.

Located on the inside of the eyelid, the Meibomian glands are small, oily and sebaceous annexes secreting lipids and proteins to cover and protect the surface of the eye and reduce the evaporation of water contained in the tears.

The lacrimal apparatus is responsible of the tear secretion, which allows the evacuation of the debris from the ocular surface and the hydration of the eye. The lacrimal fluid is continuously formed (0.1 mL/hour) by the lacrimal glands and evacuated from the eye by the lacrimal canaliculus. At the end, all of the fluid and the debris are cleared out by the nasolacrimal duct. Human tears have a mean osmolarity of 310 mOsm/kg and a tonicity equivalent to that of 0.9% sodium chloride solution [8].

### 2.2. Blood-Ocular Barriers

The blood ocular barriers are composed of the blood-aqueous and the blood-retinal barriers. They are physical barriers between the blood and the eye that has a main function in the penetration, the elimination of ophthalmic route’s drugs and the maintenance of the homeostatic control [11].

The blood retinal barrier is a posterior segment barrier forming an inner barrier in the endothelial membrane of the retinal vessel and an outer barrier in the retinal pigment epithelium [11,12]. It prevents diffusion of the drugs in the posterior part of the eye and is responsible for the homeostasis of the neuroretina, composed of nonleaky tight junctions. These junctions have a high degree of control of solute and fluid permeability. The retinal pigment epithelium controls exchange of nutriments with colloidal vessels. Retinal capillary endothelial cells and retinal pigment epithelial cells are connected to one other with tight junctions.

The blood aqueous barrier is an anterior segment barrier. It is a nano-porous (104 Å) and isotonic membrane (Dernouchamps and Heremans 1975; Dernouchamps and Michiels 1977) composed by the ciliary epithelium and the capillaries of the iris. The blood aqueous barrier produces aqueous humor and prevents access of large plasma albumin molecules and many other molecules such antibiotics for example, into the aqueous humor. The aqueous humor is secreted by the non-pigmented epithelium from the ciliary body [13]. The permeability of the blood-aqueous barrier is controlled by the osmotic pressure due to the sodium, chlorine and bicarbonate transport and by the physical-chemical characteristics of the drugs. Passages from the aqueous humor to the blood of lipophilic molecules are passive and active for hydrophilic molecules. The blood-aqueous barrier is composed of an epithelial barrier and an endothelial barrier. The epithelial barrier is composed of tight junctions between the non-pigmented ciliary epithelial cells and forms a pathway for the free diffusion of molecules. Iris vessels contain proteins similar to the epithelial tight junctions and form the endothelial barrier.

These barriers restricted the entry of drugs from systemic circulation to the posterior eye segment and conversely. Acute inflammation caused by intraocular surgery, induced ocular hypotony, and the use of inflammatory mediators can occur the breakdown of blood-ocular barrier. The reversal of this situation is made by the self-limited action of the inductive drug, the administration of anti-inflammatory or anti-hypotensive drug.

The ocular surface is directly exposed to the environment and to pathogens or allergens. It is an epithelial barrier composed of corneal epithelium connected with intercellular. These junctions are tight junctions, desmosomes, adherent junctions and gap junctions. The tears film is the first line of the entire ocular barrier. It washes the surface of the eye from the debris and protects the eye from the desiccation. Ocular inflammation, intraocular surgery, trauma and vascular disease can alter the ocular barrier.

## 3. Ophthalmic Forms

Firstly, the choice of the drug administration route depends of the target tissue. Different routes are described for the ophthalmic administration: topical ocular and subconjunctival administration are used to target the anterior segment; intravitreal and systemic administration are used to reach the posterior segment.

Two types of drug permeation after topical administration can be described: the transcorneal permeation from the lachrymal fluid to the anterior chamber and the transconjonctival and transscleral permeation from the external ocular surface to the anterior uvea-ciliary body and iris. Lipophilic drugs permeability is higher via the transcorneal route than for hydrophilic drugs because of the lipidic composition of the corneal epithelium [14]. In contrast, the transconjonctival pathway is suited to hydrophilic drugs and large molecules. Topical administration is used for the treatment of anterior chamber pathologies as inflammation, allergy, keratoconjunctivitis, infection or corneal ulceration. The topical forms must satisfy the criteria of efficacy, sterility, stability and ocular tolerance.

### 3.1. Eye Drops

Eye drops are sterile and mainly isotonic solution containing drugs or only lubricating or tears replacing solution. This conventional dosage form for ocular administration represents 90% of the marketed formulations due to its simplicity of development and production. Eye drops are cheaper than the other forms and have a good acceptance by patient [2]. Unfortunately, 95% of the drugs are eliminated with the lachrymal apparatus and the different barriers in 15 to 30 s after the instillation [14]. Moreover, a secondary eye infection may be caused by a microbiological contamination with multidoses packaging. The pH must be ideally around 7.4 which the pH of the tears [15] and the osmolarity around 310 mOsm/kg. Despite a little burning sensation after administration, responsible for lacrimation and cell desquamation, eye drops, single or multidose, are the most common dosage forms for the eyes.

However, the ocular bioavailability can be improved by increasing drug permeation through the cornea and the eye drop residence time at the eye surface. For this purpose, excipients as permeation enhancers, viscosifiant agents and cyclodextrins are used to improve the efficiency formulations [15]. Permeation enhancer modifies the corneal integrity and decreases barrier resistance [3]. Examples of permeation enhancers include polyoxyethylene glycol ester and ethylenediaminetetra acetic acid sodium salt [15]. Benzalkonium chloride is popularly used as preservative but could also plays the role of penetration enhancer due to its surfactant properties [16,17]. Viscosity enhancers by increasing the viscosity of solution allow the improvement of the residence time on the eye and the local bioavailability of the drug. To increase residence time of eye drops viscosifiant are used such as polyvinylalcohol (PVA) [18], hydroxylmethylcellulose, hydroxylethylcellulose [15]. Cyclodextrins (CD) are polysaccharides with a hydrophobic internal cavity and a hydrophilic external surface [19]. Sigurdsson et al. used CD to form inclusion complex with lipophilic molecules such as steroids or cyclosporine [20]. CD also allow the stabilization of drugs in aqueous solutions, the decrease of a local irritation after administration and the increase of the permeation of the drug through the ophthalmic barrier [21].

### 3.2. Ointments

Ophthalmic ointments are sterile, semi-solid, homogeneous preparations intended for application to the eye (conjunctiva or eyelid). Non-aqueous excipients are mainly used for this preparation and it must be non-irritating for the eye. Four types of ointment are described: oleaginous base, absorption base, water-removable base and water soluble base [22]. The oleaginous base is a lipophilic ointment, immiscible with water avoiding moisture evaporation. Composed of petrolatum and white ointment in a large amount, it can remain on skin or mucus for long period without drying out (Sterdex^®^ , Thea, Clermont-Ferrand, France ). The adsorption base may be used as emollient and contains lanolin, fatty alcohol and petrolatum (Maxidrol^®^, Norvatis, Bazel, Swizerland ). It can adsorb a quantity of water and is difficult to wash. A water-soluble base is composed only of water soluble excipients as macrogol with high molecular weight. This hydrophilic ointment is easy to wash but its use is limited due to the possible discomfort from the osmotic effect. Water removable base is an oil in water emulsion, easy to wash and easily miscible with water. It facilitates the contact between the skin and the drug but of the presence of hydrophilic surfactant (such as lauryl sulfate) in formulation can be irritating for the eye.

Unlike eye drops, this form slows down the elimination of the drug by the tears flow and increases the corneal residence time by prolonging surface time residence. Ointment application is responsible for blurred vision and its administration is advised in the evening. The packaging can be single dose or multidose and the content is limited to 5 g of preparation.

### 3.3. Hydrogels

In ocular administration, hydrogels are used to increase residence time of drugs on the eye. Hydrogels are three-dimensional water-swollen structure, composed of a viscosity agent dispersed in water or hydrophilic liquid. Hydrogels are retained in the eye and well better tolerated than ointment by patient by decreasing the side effects induced by the systemic absorption. There are two types of hydrogel, the preformed gels and the in situ gels. Gels are usually composed of hydrophilic polymers. Research focus on the development of new materials and hydrogel has many potential applications in ocular drug delivery. Applications of hydrogels were recently described in a review [23]. The main disadvantage of this form can be the quantity and the homogeneity of the drug loading in the hydrogel which can be limited, specifically in the case of hydrophobic drug. Moreover, the viscosity of gels must be stable over time to maintain the physical properties and the efficacy of the product.

The preformed gels are simple viscous solution administered on the eye. This type of polymeric gels is commonly used as bioadhesive hydrogel to improve residence time on the eye and reduce dosing frequency [2]. Mucoadhesion is the adhesion of a drug delivery system to the mucosal surface for releasing drugs in a controlled way method. Various mucoadhesive polymers were described in the literature [24,25], such as methylcellulose, hydroxylethylcellulose, sodium hyaluronate, sodium alginate, povidone, polyvinylalcohol. Sodium hyaluronate is frequently used as a bioadhesive polymer in gel formulation [26,27,28] due to it viscoelastic properties and its water retention capacity. This polysaccharide is used in the treatment of dry eye disease such as Vismed^®^ (Horus Pharma, Saint-Laurent-du-Var, France), Aqualarm^®^ (Bausch + Lomb, Bridgewater, NJ‎, USA) Hylo^TM^ (Candorvision, Montreal, QC, Canada).

In situ hydrogels are instilled as drops into the eye and undergo a sol-to-gel transition in the cul-de-sac with external changes (pH, temperature or ions). This formulation improved ocular bioavailability by increasing the duration of contact with corneal layer and reducing the frequency of administration [29]. In situ gelling delivery systems for the ocular administration of drugs improve the treatment of diseases of the anterior segment of the eye by simple, safe, and reproducible drug administration. Examples of in situ gelling polymers are shown in Table 1.

Thermosensitive gels are polymeric solutions that change from solution to gel with temperature modification. Three types of thermosensitive hydrogels can be described: negative gels, positive gels and reversible gels. The first is characterized by a decrease of the volume of the gel when the temperature increases. For the positive gels, the volume of the gel increases when the temperature increases [36]. Finally, the reversible gel [37] is characterized by a transition from solution to gel with an increase of the temperature due to a physical reticulation instead of a chemical reticulation. One of the most used polymers is poloxamer [38]; [34,39,40,41,42], a nonionic triblock copolymer composed of a central hydrophobic chain of polypropylene oxide and two chains of polyethylene oxide (Ikervis^®^, Santen, Evry, France). Several polymers can be used to accurately define the appropriate gelation temperature. For example, some researchers [43] demonstrated that the combination of poloxamer/chitosan in concentration of 16/1.0% *w*/*w* showed an optimal temperature gelation (32°C) and improved retention time. Disadvantage of thermosensitive hydrogel is the high concentration of polymer.

The pH-dependent system is induced by pH changes. pH-sensitive polymers are composed of acidic (anionic) or basic (cationic) groups. They accept or release proton and change the external pH. This change induces the swelling of the formulation and the release of the drugs. When polymers are composed of acidic groups, the solution turned to a gel by raising the pH. In contrast, polymers with basic group are converted to a gel with a pH decrease. Carbomer (Carbopol^®^, Lubrizol, Wickliffe, OH, USA) is frequently used in the formulation of in situ pH-dependent gels (Geltim^®^ LP, Thea, Clermont-Ferrand, France). For example, studies performed with a combination of carbomer (Carbopol^®^ 940) and hydroxylpropylmethylcellulose (HPMC-Methocel^®^ E50 LV, Dow Chemical, Midland, MI, USA) resulted in an improvement of the stability, non-irritability and sustained ofloxacin release (more than 8 h) [44]. Another study using carbomer 940 and different HPMC obtained a satisfactory pH between 6.0 and 7.4 for an ocular administration after gelation [45]. The hydrogel obtained enhances contact time and controlled release of norfloxacin, increased the therapeutic efficacy of the drug and reduced frequency of administration. The disadvantage of this form is the risk of damaging the surface of the eye if the pH of the hydrogel is too low.

The ion triggered system is based on a change in ionic strength of external environment. The ionic hydrogel is formed and releases its drug content after a swelling induced by the change of concentration of ions inside the solution. The cations (Na^+^, Mg^2+^, Ca^2+^) present in the tear fluid of the eye come in contact with the electrolytes of the solution and the solution turned into a viscous clear gel. For example, sodium alginate is a polymer which converts into a gel due to formation of Ca-alginate by interaction with divalent cation (Ca^2+^). Ionic polymers are often used in combination with viscosity enhancers to increase the effect. The combination of sodium alginate as ionic polymer and HPMC as a viscosity enhancer improves patient compliance due to its easy instillation in the eye [46]. In another study, this combination was used to form a pH 6.5 gel which improved the release time of the drug over a period of 10 h and is non irritating [47]. Gelrite^®^ is a linear anionic polysaccharide, a deacetylate gellan gum approved as pharmaceutical excipient. The elasticity of the gel depends of the concentration of Gelrite^®^ in the formulation. A study shows that eye contact can be prolonged up to 20 h [48]. Others prove that Gelrite^®^ in situ gels have a shelf life of more than two years and a better efficacy compared with standard eye drops [49]. This combination of different polymers is used to decrease the total polymer content in the formulation and to improve gelling properties [50]. The mixture of Gelrite^®^ and alginate solution formed a hydrogel with the optimum concentration of 0.3% *w*/*w* for the Gelrite^®^ and 1.4% *w*/*w* for the alginate. These concentrations made a non-irritant in situ gelling vehicle to enhance ocular retention for the delivery of drug [51]. Limitations of this type of gel are the possibility of interference with other ion and a low precision of the gelification process.

### 3.4. Emulsions

Emulsions are a clear, transparent and thermodynamically stable system of two immiscible fluids. This system is a dispersion of oil in water stabilized by a surfactant and sometimes a co-surfactant. There are interests for this emulsion because of the improvement of drug solubilization (hydrophilic and lipophilic) and dissolution efficiency of poorly water-soluble drugs. However, they are some limitations to this form such as a blurred vision after the instillation of the product which can decrease the patient compliance. Moreover, the homogeneity of the form is related to the uniformity of drug content and the emulsion must be stable to deliver the right dosage.

Its long shelf life, easy preparation (spontaneous formation) and improvement of bioavailability make it a potential ocular drug delivery system [52,53]. In ocular administration, micro- and nanoemulsions are privileged due to the small size of the droplets. They are structured as follow: an aqueous phase, a lipophilic phase and a surfactant phase. A co-surfactant may be required in some cases. This dispersed system has the advantages of not requiring much energy because of its spontaneously formation [54]. This carrier has natural biodegradability and can be sterilized. In 2002, FDA approved a lipid anionic (zeta potential < −40mV) emulsion containing 0.05% of cyclosporine; Restasis^TM^ (Allergan, Irvine, CA, USA) [55].

Mucosal surface of the eye is negatively charged. Cationic nanoemulsions are positively charged formulations with a nanosize structure. They are useful in prolonging the residence time of the formulation in the eye because of the electrostatic attraction of the formulation and the surface of the eye. Novasorb^®^ (Novagali Pharma, Evry, France) is a cationic (+10 mV) lipid nanoemulsion containing benzalkonium chloride or cetalkonium chloride as cationic agent [56]. Cationic agent is known to be the most toxic surfactants [57]. These surfactants are considering irritating for the skin and the eye due to their ability to solubilize lipid membrane. Formulation of cationic nanoemulsion required to find an appropriate cationic agent with high positive charge, low toxicity and good ocular acceptance. Cationic nanoemulsions containing palmatine were prepared with the emulsifying/high pressure homogenization method. The researchers obtained a particle size of 190 nm, a zeta potential of +45 mV. They demonstrated an improvement of the ocular residence time and concluded on a predominant cellular uptake and an internalization in the corneal epithelial cells [58].

### 3.5. Ophthalmic Insert

Ocular inserts are flexible polymeric materials placed in the cul-de-sac of the conjunctiva between the sclera of the eyeball and the lid. Discovered in 1971 [59], they are biologically inert, insoluble in tears fluid, sterile and non-allergic. This form was developed in order to attempt better ocular bioavailability and sustained drug action by increasing the contact time between drug and tissue of the eye. They also reduce systemic absorption and improve compliance of patients. Ocular inserts are exempt of preservative [60] and must be removed if necessary when they are no longer needed. However, they also present some drawbacks as the patient discomfort due to the solidity of this form, difficulty in placement, unintentional loss. It is also an expensive form and it can have some reluctance of the patient to use unfamiliar type of ophthalmic medication.

Different types of ocular inserts are defined: soluble inserts, bioerodible inserts and insoluble inserts. Soluble inserts are made of natural polymers (collagen), synthetic or semi-synthetic polymers (HPMC, PVA) and are degraded in the eye. Lacrisert^®^ (Idis Limited, Weybridge, UK ) is an example of commercial soluble ophthalmic drug insert. This product is used against dry eye. After its placement in the periocular space, the polymer soaked of lachrymal fluid from the tears and the conjunctiva and dissolved slowly.

Bioerodible inserts are made of biodegradable polymers (polyorthoester, polyorthocarbonate) and they do not require removal at the end of use. The polymer is gradually eroded or disintegrated, and the drug is slowly released from the hydrophilic matrix. Recently, inserts of diclofenac sodium were developed using HPMC both for the drug reservoir and for the rate controlling membrane and dibutylphtalate as plasticizer [61]. Formulation made with 3% of HPMC in drug reservoir and 3% of HPMC in rate controlling membrane increased residence time and reduced the frequency of administration. HPMC was also used in association with cyclodextrins and PVA to make ocular insert of lidocaine for topical ocular anesthesia [62]. The results revealed that lidocaine with β-cyclodextrin (βCD), 4% of HPMC and 2% of PVA have appropriate flexibility, good characteristics and the addition of β-cyclodextrins increase the drug content in the aqueous humor.

Insoluble inserts, also called ocusert, are composed of different types: osmotic systems, diffusion systems and hydrophilic contact lenses [60]. This form needs to be removed from the eye after use. The drug can be dissolved or dispersed in a reservoir. This reservoir is liquid, semi-solid, solid or can contained nanocarriers (nanoparticles). Osmotic inserts are constituted of two parts; a central part with one or two compartments surrounded by a peripheral part. Drug release occurs by the solubilization of the constituents. They generate a hydrostatic pressure against the polymer matrix that allows the release of the drug [63]. Dispersible systems are composed of semi-permeable or microporous membrane (polycarbonate, polyvinylchloride) and a central reservoir (glycerin, ethylene glycol, propylene glycol). The lachrymal fluid controls the drug release and the membrane of the system controls the rate of drug release [64].

### 3.6. Contact Lenses

Contact lenses are circulated shaped system. It is a thin, curve, round piece of transparent plastic placed directly on the surface of the eye. They are used to increase the residence time of the drug in the eye [65] and allow treating anterior eye disorders. The incorporation of the drug is achieved with methods like imprinting, simple soaking and colloidal nanoparticles [66]. Important settings of the lenses development are the preservation of the oxygen permeability and the transparency of the form. They have many advantages as the exempt of preservative and the control of the size and the shape. Although contact lenses are an alternative and promising ophthalmic drug delivery system, they are an expensive form which needs handling and cleaning. Some limitations of this form are the oxygen permeability of the lenses and it potential issue, the possibility of premature drug release or the limitation of some methodology to develop therapeutic contact lenses.

The first contact lenses were made of glass, but the use of polymethylmethacrylate allowed the development of rigid lens improving the comfort of the patient which did not let oxygen pass. Since the last three decades, contact lenses were made most of the time with silicone hydrogel [67]. They are traditionally used to improve vision defects, for cosmetic effects (change the appearance of the eye like the color) or more recently for therapeutic reasons. There are two types of therapeutic contact lenses: the scleral rigid gas permeable (RGP) lenses and the soft lenses. Scleral lenses are large, thin lenses, having a diameter from 18 mm to 24 mm. They are used in several indications [68] such as several ocular conditions [69], the correction of refractive disorders [70], provide relief on corneal irregularity [71,72,73], protection of the cornea for ocular chronic disease [74] and treatment of different ocular conditions such as glaucoma, chronic dry eye, allergies and infections [75].

### 3.7. Intraocular Injections

Intraocular injections are performed for posterior segment diseases. This technique is used in specific pathologies and requires the presence of trained and competent personnel. The surface of the eye is anesthetized during all the procedure. This technique needs a clean room, sterile materials and takes 15 to 30 min. Only solution or suspension of drug can be injected. Medications are injected through the corneal barrier, in the vitreous. Clear solutions contain antibiotic, antifungal, anticancer agent or antiviral. Avastin^®^ (Roche, Bazel, Swizerland) or Lucentis^®^ (Norvatis, Bazel, Swizerland) are commonly used in the treatment of the age-related macular degeneration. Other diseases such as the endophthalmitis, the uveitis, the diabetic retinopathy and the retinal vein occlusion are treated with intraocular injections.

### 3.8. Innovative Forms

**Figure 2:**
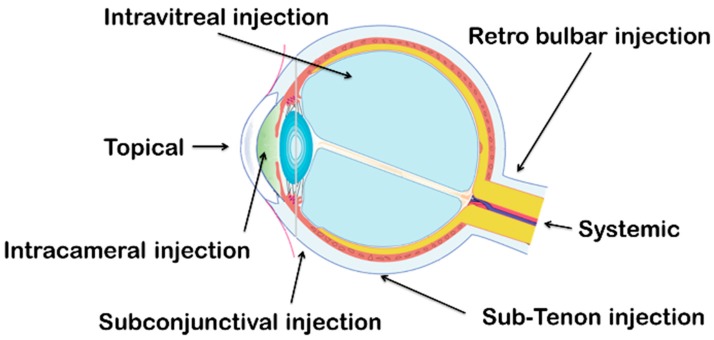
Routes of ocular administration.

For many years, researchers explored and discovered different forms for ocular administration. Among them, colloidal dispersions such as microemulsions, nanoemulsions, micro- or nanoparticles and liposomes were mainly described as innovative systems for ophthalmic delivery during last decades. They are able to penetrate the eye by the anterior or the posterior segment. These structures are presented in Figure 2.

Microemulsions are clear, transparent and thermodynamically stable systems of two immiscible fluids. This system is a dispersion of oil in water stabilized by a surfactant and sometimes a co-surfactant. Microemulsions allow the improvement of drug solubilization (hydrophilic and lipophilic) and dissolution efficiency of poorly water-soluble drugs. Its long shelf life, easy preparation (spontaneous formation) and improvement of bioavailability make it a potential ocular drug delivery system [52,53].

In ocular administration, nanoemulsion is privileged due to their small size; below 1 µm. Nanoemulsions are structured as follow: an aqueous phase, a lipophilic phase and a surfactant phase. A co-surfactant may be required in some cases. In some case, this dispersed system as the advantages of not required much energy because of its spontaneously formation [54]. This carrier has natural biodegradability; his small size allows an easy sterilization by filtration.

Nanoparticles are a nanotechnology defined as solid particles with at least one dimension less than 1 µm. These carriers have the capacity to entrapped drugs in different ways. According to the composition of the particles, there are two types of nanoparticles composed of natural or synthetic polymers, metals, lipids and phospholipids; the nanospheres and the nanocapsules [76]. Nanospheres are nanovesicles of polymeric matrix where the drug can be entrapped or attached to the surface of the particles. Nanocapsules are composed of a hydrophilic or lipophilic core surrounded by a polymeric coating. Active substances are dissolved and encapsulated in the core. Nanocarriers present many advantages; the small size and the large surface characteristic of the particles and their potential to be easily incorporated into topical formulations for ophthalmic administration with topical forms, the controlled and sustained release profiles of drugs, the spontaneous entrapment of active substance, the improvement of drug therapy and the decrease of side effects and the potential specific-site targeting [77,78]. In addition, there are some limitations; the potential particles aggregation due to their small size and their large surface area, the physical handling may be difficult in liquid and dry forms and the small size may limited the entrapment of the drug [77,79]. Moreover, due to their physical characteristics, some potential systemic toxicity can occur [80]; the systemic toxicity of nanoparticles refers to the ability of particles to adversely affect the normal physiology. From a biomedical perspective, nanoparticles toxicology reveals an interaction between the physicochemical characteristics of particles and their biological effects. The cytotoxicity of the nanoparticles can be related to the oxidative stress with the generation of reactive oxygen species or pro-inflammatory gene activation. Type of the particles (metallic substances or not), nanoparticle characteristics (morphology, size and surface) or route of administration are parameters that can induce some toxicity. Due to their small sizes, when used in intraocular way, nanoparticles could pass across ophthalmic barriers such as the trabecular meshwork leading to a systemic drug diffusion [81]. Used for topical application, nanoparticles usually do not cross corneal epithelium; Mun et al. have showed that even nanoparticles small as 21 nm do not cross neither intact cornea nor denaturated cornea [82].

Introduced in 1965 as drug delivery carriers [83], liposomes are biodegradable and biocompatible vesicular systems composed of phospholipid bilayers surrounding aqueous compartments. According to their size and their structure liposomes are in: small unilamellar vesicles (SUV) with a size ranged from 20 nm to 200 nm; large unilamellar vesicles (LUV) from 200 to 3000 nm and multilamellar vesicles (MLV) higher than 1 μm. Unilamellar vesicles are composed of one layer of lipids and multilamellar are composed of various layers of lipids. Lipophilic drugs and hydrophilic drugs are entrapped in the phospholipid bilayer and the aqueous core respectively. In ocular drug delivery, liposomes offer the advantages of a nanocarrier system with a higher biocompatibility and tolerance, and can treat both anterior and posterior segment eye diseases after topical, subconjunctival or intravitreal administration [84,85]. The surface of the vesicle can be negatively, neutral or positively charged, depending of its composition. Because of the negatively charge of the ocular mucus, the positively charged liposomes seem to be the most efficient for a prolonged adhesion to the corneal surface [86].

Niosomes are non-ionic surfactant vesicles and specific type of liposomes. With a ranged size from 10 to 1000 nm, they are biodegradable, bilayered structures stable and have low toxicity due to its non-ionic nature. Sorbitan monooleate (Span), polysorbate (Tween^®^) and cholesterol are popularly used as surfactant [87,88].

**Figure 3:**
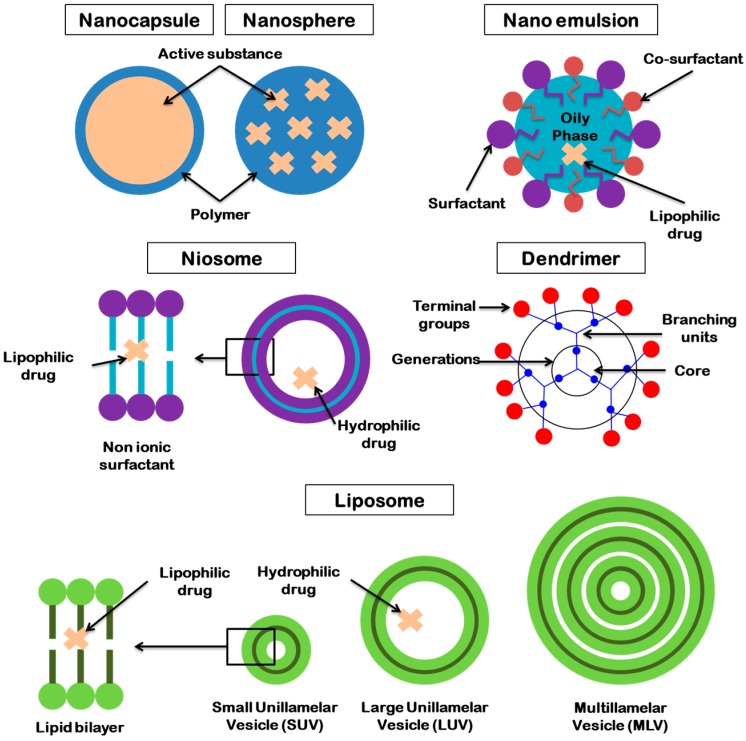
Schema of micro- and nanostructure intended for ocular drug delivery.

Dendrimers are “tree-like”, nanostructured polymers. This system is a potential carrier for ocular drug delivery due to its nanosize dimensions (1–100 nm) and its low polydispersity. They are structured as a three-dimensional globular shape (Figure 3). The core is in the center of the structure, it can be an atom or a functional molecule. The branching units are covalently bound and there are a large number of branging points regrouped in a series of radically concentric layer called generation. The terminal groups are located at the surface of the dendrimer and are functional molecules [89]. Dendrimers have lipophilic properties. New generation of dendrimers is cationic charged and potentially toxic for an ocular delivery. The old generation of anionic and neutral charged dendrimer have a higher biocompatibility of the ocular delivery [90]. Vandamme et al. formulate dendrimer with amine, carboxylate and hydroxyl surface group to increase residence time in the eye. Albino rabbit were used as an in vivo model to determine the residence time of the dendrimer in the eye and the ocular tolerance of the solution. After an instillation of 25 µL, the residence time increase with carboxylic and hydroxyl surface group. Moreover, when the dendrimer concentration increases, there is not a prolongation of the residence time, but this parameter depends of the size and the molecular weight of the dendrimer [91].

## 4. Recent Advances for Ocular Antibiotics Administration

### 4.1. Antibiotics and Ophthalmic Delivery

The first antibiotic industrially developed was penicillin, discovered by Fleming [92], which saved millions of lives and revolutionized therapies. Antibiotics are chemical substances produced naturally by microorganisms or chemically synthetized. They are used to treat or prevent infection caused by germs (bacteria or other parasites). They work by preventing bacteria from reproducing and spreading (bacteriostatic) or by killing them (bactericidal). Bacteria are unicellular microorganisms with a circular double-stranded DNA and a cell wall except for mycoplasma genus. They may be cylindric (bacilli), spherical (cocci) or spiral (spirochetes). *Streptococcus pneumoniae*, *Haemophilus* influenzae are example of bacteria that have a capsule and this encapsulation increases its virulence. Aerobic bacteria need oxygen to produce energy and growths in culture and the other bacteria are either anaerobic or facultative (can growth with or without oxygen).

The classification of the antibiotics can be done in different ways; mechanisms of action, spectrum and mechanism of action. Mechanisms of action are different from an antibiotic to another [93]; they can work on cell wall synthesis as beta-lactam (penicillin, cephalosporin), fosfomycine and glyco-, lipo- and peptides. Bacteria cells are composed of peptidoglycan and their growth is preventing by inhibiting the synthesis of this macromolecule. Aminoside, macrolide/lincosamide, chloramphenicol and tetracycline are active on protein synthesis from the bacteria. They inhibit the 30S ribosome subunit (aminoside and tetracycline) or the 50S ribosome subunit (macrolide/lincosamide, chloramphenicol), responsible for the binding of the tRNA to the receptor site on mRNA. Other antibiotics inhibit folate synthesis as sulfamides, and dihydrofolate reductase inhibitor. They block nucleotides, lipids and amino acid synthesis from bacteria cell. Finally, fluoroquinolone, sulfamide and rifampicin are working on DNA and RNA synthesis. Antibiotics can also be classified by their spectrum; broad spectrum antibiotics affect numerous infections, including gram-negative and gram-positive bacteria, and narrow spectrum antibiotics are active against a selective type of bacteria. Among broad spectrums antibiotics we can find amoxicillin (beta-lactam), tetracycline, cephalosporin, chloramphenicol, and erythromycin (macrolide). Short spectrum antibiotics group are composed of penicillin-G, vancomycine (glycopeptide).

Antibiotics can be bacteriostatic as tetracycline, chloramphenicol, and erythromycin. Cephalosporin, erythromycin and penicillin are examples of bactericidal antibiotics. Bacteriostatic antibiotics do not work if a bactericidal antibiotic is given concurrently. To avoid interaction between these drugs, they have to be alternatively administrated and not in combination [94].

Eye infections must be treated by appropriate and safe use of antibiotics. Antibiotics can be administrated by several routes (oral, parenteral, local) and the most appropriate administration depends on the area of the eye to be treated. The anterior segment (cornea, conjunctiva) is frequently treated with local administration. Topical administration is used for eye drops, ointments or gels; each form presents a main advantage like an immediate action for eye drops, a decrease of the administration frequency for gels or an increase of the drug biodisponibility for ointments. The intraocular (intravitreal, intracameral) administrations lead to a greater intraocular concentration of antibiotics than any other administration. Intravitreal injections are used as prophylaxis or curative treatments of endophthalmitis with combination of vancomycin and ceftazidimeb for example [95]. Subconjunctival and retrobulbar administrations are periorbital administration. Subconjunctival is used to achieve high concentration of drugs and large size molecules or the administration of drug with low bioavailability by the topical way. Retrobulbar injections are usually used for the treatment of optic neuritis. Generally, subconjunctival route allows achieving equal or higher drug concentration than retrobulbar injections [96].

Because of the ocular barriers, the targeting of the posterior segment (retina, choroid, and sclera) always requires systemic administration (oral, parenteral). Oral administration is easy to develop and to deliver to the patient, but this way of administration is limited by the antibiotics bioavailability; only low molecular weight and lipophilic drugs can cross the blood barriers and the ocular barriers. Systemic toxicity and safety have to be considered for an oral administration with an ocular response [14]. Parenteral administration is used for preseptal cellulitis, orbital cellulitis, dacryocystitis, or in adjunction to others treatments in the ocular adnexa, orbital and periorbital tissues [97]. However, parenteral route is not the most common administration way for the treatment of ocular diseases.

Antibiotics usually have a short half-life and need repeated administrations. Using antibiotics requires knowledge of the pharmacokinetic and the toxicity of the drug for the different routes of administration. Due to their low solubility, molecules such as penicillins, cephalosporins and aminoglycosides penetrate the eye with great difficulty. In dermal application, penicillin is highly allergic and causes skin rashes and allergic sensitivity. Via oral route, tetracyclines present major side effects toward intestinal microflora. Modern betalactams and aminoglycosides have to be injected because of their low bioavailability by oral route. All of these side effects favor the ophthalmic administration to increase the tolerance of the active substance.

### 4.2. Recent Advances in Ocular Delivery of Antibiotics

#### 4.2.1. Improvement of Drug Dissolution and Stability Using Cyclodextrins

Cyclodextrins (CD) were discovered in the 1900 and more recently used in ocular drug delivery. They are cyclic oligosaccharides with an inner lipophilic cavity and a hydrophilic outer surface. They are used as solubilizer, drug stabilizer, permeation enhancers, separation agent in HPLC or catalyst and additives. These excipients increase solubility and stability of drugs, prevent side effects as irritation and discomfort [98]. Cyclodextrins should be non-irritating, non-toxic, well tolerated, inert and enhance permeability of the drug through the corneal mucosa. CD can be used in particles (nanosphere, microsphere, liposome) [99].

Hydroxylpropyl-β-cyclodextrin (HPβCD) was used to create a complex with ciprofloxacin in order to formulate eye drops. The inclusion complex showed a better stability, an ocular tolerance and a higher biological activity in comparison to marketed eye drops and simple aqueous solutions [100]. The same combination increased the solubility of ciprofloxacin from 3-fold at pH 5.5 and 2-fold at pH 7.4. The authors noticed that the complex at pH 5.5 had a higher stability after two months of storage than the complex at pH 7.4. The stability of the drug increased with the HPβCD and the complex improved the in vitro release of the drug [101].

Novel βCD polymers are incorporated at complexes with rifampicine, novobycin or vancomycin into a hydrogel showed a slower release of the drug compared to the dextrose-based gels. The study demonstrated that the larger and more hydrophilic drugs had release profiles less altered than small hydrophobic drugs [102].

#### 4.2.2. Contact Lens for Antibiotic Delivery

Contact lenses were used as drug reservoir or support for the active ingredient in antibiotic ocular delivery. Initially, they are used as ophthalmic system to correct vision. The scleral RPG (Rigid Gaz Permeable)) lenses trap a tear reservoir, which can be used as a drug container. It prevents tear evaporation or adhesion from mucus filament in the cornea, has a potential cornea healing or hydrates the cornea in severe case of dry eye disease [103]. It prevents eyes of the patient from exposure to their irregular cornea and the reservoir can contain some artificial tears needed to lubricate the surface of the eye. In the toxic epidermal necrosis and Steven-Johnson syndrome, wearing scleral lens improves the relieving symptoms. The liquid reservoir of this lens can contain some drug as topical corticosteroids and cyclosporine [104]. More recently, a study describes the in vivo release of ofloxacin from a scleral lens in rabbit with keratitis. This preclinical study assesses local tolerance and intraocular diffusion of the antibiotic administrated by a contact lens. The authors found a higher level of drug in aqueous humor and cornea than those reported with other administration route [105].

Soft contact lenses are often composed of hydrogels, like hydroxyethyl polymetacrylate hydrogel [106]. More recently the use of silicone hydrogel was described offering more oxygen transmission than the standard hydrogel lenses. The most common preparation technique of contact lenses for controlled drug delivery is the “soaking” technique. Briefly, lenses are immersed in an antibiotic solution. The uptake and release of antibiotics were explored to compare the ability of different commercial lenses to release fluoroquinolone; 1-Day Acuvue^®^ (Johnson & Johnson, New Brunswick, NJ, USA) Medalist^®^ (Bausch & Lomb, Rochester, NY, USA) and 14UV. There were soaked in fluoroquinolone solutions during different times. In conclusion, the higher uptake of drug was for the 1-Day Acuvue^®^ lens and the release rates were slower for the 1-Day and the Medallist^®^ than for the 14UV, but the most practicable system was the 1-Day Acuvue^®^ [107]. These conclusions were previously exposed by Hehl et al. [108]. Fluoroquinolone and aminoglycosides loaded contact lenses (gentamycin, kanamycin, tobramycin, ciprofloxacin, ofloxacin) were studied to improve the ocular penetration of topically applied drugs. They used Acuvue^®^ contact lenses, soaked in the different antibiotic solutions. In conclusion, kanamycin was not able to cross the corneal barrier and only gentamicin, ciprofloxacin and ofloxacin produced bacteriostatic concentrations in the aqueous humor.

Derivate from the soaking technique, the supercritical CO_2_ impregnation/dispersion method is also explored due to its non-toxicity, its low surface tension of the polymer and its high diffusivity [109]. This technique permits to prepare commercial soft contact lenses such as FocusDailies^®^ (Novartis, Basel, Switzerland), Proclear^®^ Compatibles (CooperVision, Lake Forest, CA, USA), Frequency^®^ 55 (CooperVision, Lake Forest, CA, USA) and SofLens^®^ 59 Comfort (Bausch & Lomb, Rochester, NY, USA). The study concludes that this drug delivery system obtained with the supercritical solvent impregnation can be viable, safe and efficient such as the impregnated lens obtained with the soaked method [110]. The molecular imprinting technology during the lens manufacturing forms, in the contact lens, structures like pockets, which are memorizing the spatial feature and the bonding preferences of the drug [111]. A norfloxacin (quinolone) delivery system with imprinting method was described using different ratios of drug and acrylic acid. With the most efficient ratio (1:4), they demonstrated that the high affinity binding point allows to make lenses able to control drug delivery release from several hours to days [112]. The development of drug-soft contact lenses with polymyxin B and vancomycin against *Pseudomonas aeruginosa* demonstrated a good biocompatibility of the two hydrogels but imprinting effect only exhibited with polymyxin B [113].

#### 4.2.3. Ocular Inserts for Antibiotic Delivery

Ocular insert is solid or semi-solid preparation placed in the cul-de-sac to deliver a controlled flow of drug. The use of ocular insert for antibiotic delivery was also described in the literature. In 1980, some researchers studied the in vitro and the in vivo release of antibiotics such as erythromycin and erythromycin estolate from matricial ocular inserts. They discovered that when the water content of the hydrogel insert is more than 30%, the elution rate of a low aqueous solubility drug is constant [114]. In the same time, drug-inserts with copolymers of *N*-vinylpyrrolidone tested completely suppressed the chlamydia trachomatis infection in the monkey eyes [115]. In a study, macrolide antibiotics (erythromycin) and penicillin were evaluated as a potential ocular drug delivery system in an antibiotic-impregnated collagen insert. The system with the erythromycin and the soluble collagen produced the most interesting system due to his sustained drug delivery [116]. To treat external ophthalmic infections, a combination of the aminoglycoside, gentamicin sulfate, and dexamethasone phosphate in a soluble insert was developed. The matricial insert was composed of HPMC, ethylcellulose and carbomer. This new form prolonged the release of gentamycin above the minimum inhibitory concentration value (MIC) of 4μg·mL^−1^ for nearly 50 h. The dexamethasone side effects caused by repeated instillation were avoided and the compliance improved [117].

Many fluoroquinolones were used as drug for ocular controlled delivery in an insert. For example, ofloxacin was studied in erodible insert with poly(ethylene oxide) (PEO). After application of the insert (6 mm of diameter and 20 mg of weight), a gel formed. The aqueous maximum concentration was higher than the commercial eye drops. Bioavailability improved due to the mucoadhesion of PEO and tear fluid viscosity [118]. This gelling system was explored with different molecular weight of PEO (from 200 to 2000 kDa). The molecular weight of PEO had huge influence on the erosion time consequently on the transcorneal absorption, the gel residence time, the drug release, the drug residence time in the aqueous humor at concentration higher than MIC. The optimal mucoadhesion was for the 400 kDa PEO. The 400 kDa PEO and 900 kDa PEO have some potential for a topical treatment in endophthalmitis [119]. The in vitro release and the ocular delivery of ofloxacin in chitosan microspheres and insert were explored by the same researchers. The microspheres were added to the insert formulation to evaluate their effects on drug release mechanism from the insert and the drug penetration into the aqueous humor of the rabbit eyes. This addition produced structural changes, accelerating the erosion of the insert and the release of the drug. In conclusion, chitosan microspheres enhanced the transcorneal permeability of the drug [120]. More recently, inserts with ofloxacin encapsulated in nanolipid carriers showed a preocular retention up to 24 h and a maximum concentration in aqueous humor increased six times in comparison with the commercial. Keratitis in rabbit’s eyes were healed in 7 days [121].

Other fluoroquinolone-inserts, such as pefloxacin, were developed. They were used in bacterial conjunctivitis and were a reservoir type ocular insert. Eudragit^®^ (Evonik, Essen, Deutschland) is copolymers derived from esters of acrylic and methacrylic acid. Different ratios of Eudragit^®^ RS100 and RL100 (ethyl prop-2-enoate methyl 2-methylprop-2-enoate trimethyl-[2-(2-methylprop-2-enoyloxy)ethyl]azanium chloride) were studied. The ratio 4:1 (RS/RL) allowed a drug release from 90-98% within 48 to 120 h. This optimized formulation remained stable and intact at room temperature and provided the desired drug sustained release in vitro for 5 days [122]. Ciprofloxacin drug reservoir inserts were studied to achieve once a day administration. A hydrophilic polymer, gelatin, was used in drug reservoir and the rate controlling membrane was made by hydrophobic ethylcellulose. This form showed an increasing residence time in the eye, a sustained drug release, a decreasing frequency of administration and improved compliance of the patient [123]. These conclusions are supported by the in vitro and in vivo studies revealing the efficacy of the formulation [124].

In another study, Pawar et al. prepared an ocular insert of moxifloxacin and PVA by the film casting method. The soluble insert obtained (5.5 mm of diameter) was coated with different Eudragit^®^ (S-100, RL-100, RS-100, E-100 or L-100) and cross-linked by CaCl_2_. The mucoadhesion time and the drug content were found satisfactory. The coating and the cross linking extended drainage from insert and the formulation with Eudragit^®^ RL100 showed maximum drug penetration [125].

Macrolides were also studied in ocular insert. Azithromycin ocular inserts were formulated and evaluated. The polymer HPMC was used as drug reservoir and the Eudragit^®^ RL100 as rate controlled membrane. The concentration of 1.5% HPMC and 3% Eudragit^®^ RL100 was found to be optimized formulation. It controlled release over a 12 h period, had a better ocular tolerability and improved ocular bioavailability in ocular infections [126].

#### 4.2.4. In Situ Gelling Systems for Antibiotic Delivery

Some antibiotics were studied in different in situ gelling systems during the past two decades to improve patient compliance by: prolonging and controlling drug release, prolonging corneal contact time and enhancing ocular bioavailability. Different in situ gelling systems are used in ocular drug delivery as the thermosensitive, the ion-activated and the pH sensitive gelling system.

Different concentrations of active substance in the formulation allowed screening the efficiency on referential bacteria as *Staphylococcus aureus*, *Pseudomonas aeruginosa* or *Escherichia coli*. In a study, various concentrations of clarithromycin or levofloxacin in ophthalmic gels were tested. Two drops of each gel were administered four times per day during 4 days. The 0.25% clarithromycin ophthalmic gel had a better action again *Staphylococcus aureus* than the 0.1% clarithromycin ophthalmic gel [127].

Different excipients are used for the formulation of in situ gelling systems in order to control the mucoadhesion force and the viscosity of the formulation. HPMC is a viscosity enhancer commonly employed in gel formulation. The combination of alginate as ionic-induced gelation agent and HPMC with gatifloxacin demonstrated a higher efficacy than the alginate alone. The mixture could be used as an in situ gelling system to improve compliance of patient and increase ocular bioavailability [128]. These conclusions were confirmed by a recent study testing HPMC and sodium alginate in a pH induced gelation system developed for a ciprofloxacin ocular gel [46]. In some cases, the addition of HPMC and methylcellulose is used to increase the viscosity of the gel and decrease the concentration of carbomer in the formulation. This pH or ionic sol-in-gel transition system with ciprofloxacin, used in corneal ulcer and corneal infection, allowed prolonging the antimicrobial effect against bacteria for instance *Escherichia coli*, *Staphylococcus strains* and *Pseudomonas aeruginosa* [129].

Ciprofloxacin was also tested alone in poloxamer-based thermosensitive gel. The combination of poloxamer (407 and 188) and HPMC or HEC, as bioadhesive agents, allowed formulating an in situ gelling system with a gelation temperature between 28 and 34°C. The addition of poloxamer 407, HPMC and HEC improved the bioadhesion force, the viscosity of the formulation and decreases the in vitro drug release [130]. Moreover, the elastic properties of the ocular gelling systems allow the limitation of drug ocular drainage. Combination of poloxamer 401 and 188 with sodium alginate and xanthan gum were also explored with the moxifloxacin. The increase of the mucoadhesive polymer concentration decreased the rate of drug release. The thermoreversible mucoadhesive gels obtained have a pH of 6.8 to 7.4, were safe and sustained ocular delivery of moxifloxacin [42]. More recently, different polymers; polyox (pH sensitive agent), poloxamer (a temperature-sensitive gelling agent) and sodium alginate (an ion-sensitive gelling agent) were tested in combination with HPMC as viscosity enhancer. The in vivo assays showed sustained release of moxifloxacin hydrochloride over 8 h and the formulation were therapeutically efficient, stable and non-irritant [131]. The combination of sodium alginate and methylcellulose in an ion-sensitive gel confirmed this conclusion with a sustained release of sparfloxacin for a period up to 24 h with no ocular damage [132]. The bioavailability of pefloxacin was increased by the addition of carbomer and methylcellulose. This combination increased the gel strength. The 0.18% pefloxacin gel showed a drug level in the aqueous humor above the MIC-values over a period of 12 h compared to the 0.3% commercial eye drops indicating that the developed form is better considering this parameter. This mixture showed a better ability to retain the drug than the carbomer or methyl cellulose solutions alone [133].

An ion activated in situ gelling system of gatifloxacin showed a higher bioavailability and a longer residence time in the eye by microdialysis. Compared to conventional eye drops, this system could be viable as a potential ocular drug delivery [134].

In another study, a Gelrite^®^ in situ ophthalmic gelling system was compared to Vigamox^®^ (Alcon, Fort Worth, TX, USA) commercial eye drop for the local administration of moxifloxacin. They concluded that compared to the eye drop, higher amount of moxifloxacin was retained in the aqueous humor. Against the bacterial corneal inflammation, they had a major improvement after four days compared to seven days for the conventional eye drops [135].

#### 4.2.5. Colloidal Systems for Antibiotic Delivery

Colloidal systems are popularly employed in the development of formulation for the treatment of ocular diseases (Table 2). 

They have many advantages; prolonging the residence time of the drug on the surface of the eye, sustained release, increasing the bioavailability of the drug. The dosages’ forms included microemulsions, nanoemulsions, nanoparticles, liposomes or niosomes (Figure 3) [5,136].

##### Microemulsions for Antibiotic Delivery

Microemulsions are colloidal systems kinetically stable. They are used for their ability to deliver both lipophilic and hydrophilic drugs and to increase the bioavailability of active substances. Tween^®^ 80 (polyoxyethylene sorbitan monooleate) and Span^®^ 20 (sorbitan monolaurate) are mainly used as a non-ionic surfactant and co-surfactant for microemulsion formulation. Tween^®^ 80 is recognized as a practically non-irritating and non-toxic surfactant for ophthalmic use [137].

Lv et al. studied the stability of microemulsion containing 0.3% of chloramphenicol for the treatment of trachoma and keratitis. The organic phase is composed of butanol, isopropyl palmitate and isopropyl myristate and the aqueous phase is composed of water. They concluded with an improvement of the stability of the drug after three months compared to classical chloramphenicol eye drops. Chloramphenicol was in hydrophilic shells of microemulsion drops [138]. This improvement of stability was confirmed by another study using microemulsion for the ocular delivery of moxifloxacin for the treatment of bacterial keratitis. Droplet sizes were below 40 nm and exhibited a sustained drug release profile. The in vivo study showed a greater antimicrobial activity on bacterial keratitis in rabbit eyes than the commercial eye drops [139].

Üstündag-Okur et al. studied the addition of ethanol as co-surfactant, Tween^®^ 80 as surfactant, oleic acid as oil phase and sodium chloride in water as aqueous phase as a promising strategy for ocular drug delivery. The preocular residence time was higher with the microemulsion than the solution. The authors studied the effect of the addition of 0.75% chitosan oligosaccharide lactate (COL) in microemulsion on ofloxacin ocular penetration compared to a simple microemulsion (without COL) and a solution of ofloxacin. They observed that the permeation rate was lower with COL microemulsion than the formulation without COL. However, the COL microemulsion had a slower release of ofloxacin and a higher antimicrobial activity than the simple microemulsion. The MIC values were the same for the two microemulsions [140]. The combination of Tween^®^ 80 and the Transcutol^®^ P (diethylene glycol monoethyl ether) (Gattefossé, Saint-Priest, France) as a co-surfactant with 0.3% of gatifloxaxin formulated an oil-in-water microemulsion was explored for the intraocular drug delivery. Zeta potential ranged from +15 to +24 mV and the droplet size ranged from 51 to 74 nm. The optimized formulation, composed of 10% isopropyl miristate, 10% Tween^®^ 80, 10% Transcutol^®^ P and 70% deionized water, showed a better stability, adherence to corneal surface, permeation rate of gatifloxacin and tolerance than the commercial eye drops, Zigat^®^ (FDC Limited, Maharashtra, India). However, the transcorneal permeation of gatifloxaxin using commercial eye drops was higher during the first hour than the microemulsion due to the un-ionized forms of the drug. Finally, the developed formulation increased intraocular penetration of the drug and was a promising alternative to the eye drops [141].

##### Nanoemulsions for Antibiotic Delivery

Many studies describe the use of nanoemulsion in ocular administration. Their small size and god tolerance by the patient are advantages for an effective treatment. Unfortunately, this form is sparsely described in the literature in association with antibiotics.

Researchers explored a mucoadhesive cationic nanoemulsion of dexamethasone and polymyxin B. The innovation was in the use of a positively charged drug and preservatives to achieve mucoadhesion of cationic emulsion. The lipid phase was composed of dexamethasone 0.5% (*w*/*w*), Lipoid^®^ S100-Eutanol^®^ G (30%:70%) (soy phosphatdylcholine-octyldodecanol) (BASF Corporation, Ludwigshafen, Deutschland)) and the aqueous phase contained polymyxin B 0.1% (*w*/*w*), cetylpiridium chloride 0.01% (*w*/*w*) and glycerol 2.6% (*w*/*w*). The pH of the formulation was 5.31, droplets size was below 200 nm, and zeta potential ranged from 11 to +9 mV and the emulsion was stable after six months at room temperature and +4°C. The in vitro study demonstrated the non-cytotoxicity of the nanoemulsion and its ocular potential application as viable alternative to commercial solutions [142].

##### Nanoparticles and Microparticles for Antibiotic Delivery

Nanoparticles were explored in many cases of eye diseases. With their ability to cross the ocular tissues [143] without any influence on cornea, iris or retina, they are promising technology for increasing the therapeutic efficacy of ophthalmic therapies [144].

Das et al. studied polymeric nanoparticles composed of Eudragit^®^ RL100 and prepared by the nanoprecipitation method for ophthalmic delivery of amphotericin B against *Fusarium solani*. The particles had a size ranged from 130 to 300 nm, a positive zeta potential and encapsulation efficiency from 60% to 80%. They showed no signs of eye irritation and were stable for two months at +4°C and room temperature [145]. Other authors confirmed this conclusion. The combination of Eudragit^®^ RL100 and Pluronic^®^ F108 (BASF Corporation, Ludwigshafen, Deutschland) formulated small positive particles (below 500 nm) with no significant chemical interaction between the polymer and the drug. They noticed that changing the pH of the external phase of nanoparticle suspension increased the encapsulation efficiency of sulfacetamide [146].

Ibrahim et al., developed Eudragit^®^ RL100 / RS100 nanoparticles of coated with hyaluronic acid as bioadhesive polymer, to extend the release of gatifloxacin and prednisolone (glucocorticoid) compared to the free drug and to improve the patient compliance. The authors demonstrated that the increase of drugs:polymers ratio improved the drug encapsulation efficiency and the increase of Eudragit^®^ RS100 amount decreased the release efficiency values. The particles had a size ranged from 315 nm to 473 nm and showed better bioavailability of drugs in the aqueous humor and corneal tissue than the marketed eye drops [147]. In another study, a 50:50 (*w*/*w*) ratio of Eudragit^®^ RS100 and RL100 was tested with Tween^®^ 80 and poly(vinyl alcohol) (PVA) to improve the residence time of the gatifloxacin. The nanoparticles were prepared via the double emulsion technique or the nanoprecipitation method. The particle size was higher with the double emulsion technique and the Tween^®^ 80. The optimized positive nanoparticles had a gatifloxacin encapsulation efficiency of 46%, a prolonged release rate of gatifloxacin and prolonged antimicrobial effects against *Escherichia coli*, *Staphylococcus aureus* and *Pseudomonas aeruginosa* [81].

Poly(lactic-*co*-glycolic acid) (PLGA) is a copolymer widely used in medical and pharmaceutical applications. It is a biodegradable excipient evaluated, for example, in a rifampicin microparticulate system for an intraocular injection in order to prevent endophthalmitis during cataract surgery. The in vitro release of rifampicin and its antibacterial assessment were explored. The PLGA microparticles showed a sustained release profile of rifampicin in vitro and bactericidal effect against *Staphyloccocus epidermidis* mainly involved in endophthalmitis [148]. Similar PLGA microparticles prepared by w/o/w emulsion-diffusion method were studied for the vancomycin delivery. Depending of formulation parameters, microparticles have a negative zeta potential and a size ranged from 1.6 to 11.8 μm. The release of the vancomycin in the first 24 h was around 90% [149].

Another study explored a sparfloxacin nanoparticle system with PLGA as polymer and PVA as stabilizant. The negatively charged particles had a size from 180 nm to 190 nm and showed non-irritant properties. In vivo study and gamma scintigraphy exploration suggested that there was no drug in systemic circulation, an increase of the precorneal residence time and of the sparfloxacin ocular penetration [150]. The PLGA gave the same size and zeta potential results and an entrapment efficiency of 85% in combination with levofloxacin. Images of scintigraphy showed a good spread and good retention of the drug on the cornea. Nanoparticles retained the drug for a longer time and allowed slowing down the drain out of the drug from the eye compared to the marketed formulation. Moreover, the in vitro release showed an initial burst followed by a slow drug release over a period of 24 h [151]. Another study confirmed this in vitro conclusion. Clarithromycin loaded PLGA nanoparticles were prepared via the nanoprecipitation method with different ratio of drug:polymer (1:1, 1:2, 1:3). The negative particles obtained had a size below 300 nm and an entrapment efficiency of clarithromycin from 57% to 80%. Under encapsulated form, he drug crystallinity was decreased and the authors demonstrated that a dosing at 1/8 concentration in the particles of the intact drug is more effective against *Staphylococcus aureus* than the free drug [152].

More recently, a study developed doxycycline hyclate loaded nanoparticles prepared via the emulsion cross-linking method to improve precorneal residence time and drug penetration. Gellan gum, Aerosol^®^ OT (dioctyl sodium sulfosuccinate) (anionic surfactant) (Cytec Solvay Group, Woodland Park, NJ, USA) and PVA composed the particles. They had a size ranged from 331 nm to 850 nm and entrapment efficiency of doxycycline from 45% to 80%. *Ex-vivo* studies showed a higher sustained release from particles in both *Staphylococcus aureus* and *Escherichia coli* strains than the doxycycline hyclate aqueous solution. The authors noticed that formulations were non-irritating for the eye, inhibited bacterial growth and were a potential drug delivery system for ocular bacterial infections [153].

Chitosan is a natural mucoadhesive, biocompatible, positively charged polymer. In combination with sodium alginate and Pluronic^®^ F127, nanoparticles demonstrated a prolonged topical ophthalmic delivery of gatifloxacin. They are positively charged (+18 to +48 mV) and had a size from 205 to 572 nm. The in vitro studies showed a fast release for the first hour and non-Fickian diffusion process for the gradual drug release during the next 24 h [154]. In addition, Silva et al. developed mucoadhesive chitosan, sodium tripolyphosphate particles for daptomycin ocular delivery prepared by the ionotropic gelation method. Particles exhibited small size (200 to 500 nm, polydispersity index from 0.1 to 0.2) and round-shape. They obtained a total release of the drug within 4 h and the incubation of the particles with lysozyme positively affected their mucoadhesive properties [155]. More recently, a study demonstrated that the combination of chitosan and sodium alginate as a mucoadhesive coating for nanoparticles (size from 380 to 420 nm, entrapment efficiency from 79% to 92%) allowed an epithelial retention of daptomycin compared to the solution of the free drug [156].

Solid lipid nanoparticles (SLN) were considered as promising carrier for ocular drug delivery strategies. There are characterized by a physiological lipid core surrounded by an aqueous phase and stabilized by surfactants. Hydrophilic and lipophilic drugs are entrapped in this particles presenting the advantages of a good safety; a large-scale industrial and sterilizable production feasibility [157]. SLN for example, were studied for the ocular administration of gatifloxacin, with stearic acid alone or a stearic acid/Compritol^®^ (Glyceryl behenate) (Gattefossé, Saint-Priest, France) mixture and poloxamer 188 as surfactant. The authors concluded to a higher average size, entrapment efficiency and lower crystallinity for the lipid matrix SLN (composed of stearic acid/Compritol^®^) than for the stearic acid alone SLN. In addition, the formulations had a positive zeta potential and were physiologically tolerable by the eye [158]. A Box Behnken statistical design with 3 variables and 3 responses were used to optimize the development of a gatifloxacin SLN. The cationic carriers were composed of lipids (stearic acid and Compritol^®^ or stearic acid and Gelucire^®^ (Gattefossé, Saint-Priest, France), poloxamer 188 and sodium taurocholate and prepared by o/w-emulsion method. SLN size ranged from 250 nm to 305 nm had a zeta potential from +29 to +36 mV and gatifloxacin entrapment efficiencies from 47% to 79%. The authors studied the corneal permeation of drug on a freshly excised goat cornea and its effect on corneal hydration level compared to Gate^®^ (gatifloxacin 0.3%) (Ajanta Pharma, Maharashtra, India) eye drops. They concluded to an increase of 3.37-fold for the bioavailability of the drug, 2.34-fold for the half-life and 1.09-fold of concentration of drug in the aqueous humor in favor of the SLN. The authors suggested that, with no signs of irritation, the formulations could prolong the residence time in the eye and enhance the bioavailability of the drug [159,160].

A Box Behnken experimental design with 3 variables (stearic acid, Tween^®^ 80 and sodium taurocholate concentrations) and 2 responses was also performed to optimize the preparation of levofloxacin SLN. With a particles size of 238 nm and a entrapment efficiency of 79%, the optimized formulation showed a flux of 0.2493 μm/cm/h through excised goat cornea, a prolonged drug release and an equivalent antibacterial activity against *Staphylococcus aureus* and *Escherichia coli* compared to the marketed eye drops [161].

In another study, the double emulsion method was used to prepare vancomycin SLN and enhance the ocular penetration of the drug and its residence time in the eye. The molar ratio lipid:drug of 1:1 (glycerylpalmitate:vancomycin) with low molecular weight of PVA allowed nanoparticles of 278 nm, a zeta potential of −20 mV and an entrapment efficiency of 20%. The authors concluded that the encapsulation efficiency of the drug was not enough due to the high water solubility of the drug, clinical application are consequently not possible [162].

Finally, intraocular delivery of tobramycin with stearic acid SLN was studied for targeting the posterior segment and the inner parts of the eye against *Pseudomonas aeruginosa*. The vesicles had a size of 80 nm, a polydispersity index of 0.15 and a zeta potential of −26 mV. They demonstrated a higher concentration of drug in the ocular tissue with a topical administration compared to the commercial Tobral^®^ (Alcon, Fort Worth, TX, USA) eyedrops and a slow and constant release of tobramycin [163].

##### Liposomes for Antibiotic Delivery

Phosphatidylcholine (egg and soy) (PC) and cholesterol (CH) are lipids popularly used in liposomes preparation. To provide long-term drug delivery without avoiding systemic drug exposure, a study explored a ciprofloxacin hydrochloride liposomal system. Different molar concentrations of CH were studied, and it appeared that this parameter influenced the particle size, the drug entrapment efficiency and its release. The sizes of the particles ranged from 2.5 to 7.2 μm. Ciprofloxacin had a fast release profile during the first hours, then the drug release followed the Higuchi diffusion model. The authors showed that the drug release was controlled by the drug concentration during the first 10 h and, after 10 h, by the concentration of CH [164]. More recently, Chetoni et al. compared the efficacy of distamycin a liposomes to a simple solution, for Herpes simplex virus treatment. The combination of PC and CH was used. Using PC/CH liposomes, the authors showed that the ocular tissue toxicity was reduced with this formulation and that the ocular bioavailability and retention into the cornea were increased [165].

Another study investigated the influence of different molecular weights and concentrations of chitosan for the coating of ciprofloxacin liposomes. Despite a lower encapsulation efficiency of the drug, coated liposomes improved ocular penetration and antimicrobial activity of ciprofloxacin. In vitro studies showed that the formulation inhibited the growth of *Pseudomonas aeruginosa* in rabbit’s eyes for 24 h. In addition, a higher concentration and molecular weight of chitosan increased the mucoadhesion properties of the liposomes [166]. More recently, a study with the combination of chitosan, liposomes and ciprofloxacin hydrochloride concluded to the improvement of the bioavailability of the drug. The liposomes were composed of PC, CH at different ratio and stearylamine. Optimized formulation obtained with a ratio PC:CH of 10:0 showed the better entrapment efficiency of ciprofloxacin of 39% and an in vitro release after 8 h of 79% [167].

To increase the contact time between the drug and the surface of the eye, the liposomal gels showed great potential. MLV were formulated with lecithin and PC in a bioadhesive poyl(vinyl alcohol) and polymethacrylic acid gel. This formulation aimed to minimize the dilution effect of tear in the conjunctival sac and ensured a steady and prolonged drug release. The liposomal encapsulation of the ciprofloxacin extended the in vitro release of the antibiotic [168]. Hosny et al. developed a hydrogel of liposomal suspension for the ophthalmic delivery of ofloxacin. The use of an ophthalmic solution requires a frequently instillation in the eyes and due to its pH dependent solubility ofloxacin tends to deposit on the eye surface. MLV and reverse-phase evaporation vesicle (REV) are formulated. MLV have better entrapment efficiency and the liposomal hydrogel enhanced the transcorneal permeation 7-fold more than the aqueous solution. Authors also demonstrated that a thermosensitive prolonged release liposomal hydrogel provided in vitro ocular bioavailability through albino rabbit cornea. This formulation allowed minimizing the frequency of administration and decreased ocular side effects of ofloxacin [169].

Gatifloxacin and ciprofloxacin were studied with the same liposomal hydrogel to enhance transcorneal permeation. Liposomes are composed of phosphatidylcholine and CH, stearylamine or dicetyl phosphate, both used to respectively provide to liposomes either a positive charge or a negative charge. Liposomes were dispersed in a Carbopol^®^ 940 (carbomer) hydrogel. Optimal entrapment efficiency was obtained for the ratio 5:3 (PC:CH) and the best release of hydrogel and transcorneal penetration was obtained for the ratio 5:3:1 (PC:CH:stearylamine). In addition, the increase of CH content above this limit decreased the entrapment efficiency and the positively charged liposomes entrapped more drug than the negatively charged liposomes. They concluded that the hydrogel ensured steady prolonged transcorneal permeation and improved the ocular bioavailability of the antibiotics [170,171].

Intravitreal injections are mainly used as conventional therapy for bacterial endophthalmitis. To improve these treatments and prolong intravitreal therapeutic concentrations of antibiotics, these drugs were entrapped in liposomes. For example, Zeng et al. encapsulated amikacin into liposomes with an entrapment efficiency of 91%. The half-time release of the drug from liposomes in PBS was 84.8 h. This formulation prolonged half-life of the drug in the vitreous and the pharmacokinetic analysis suggested that in severe case of endophthalmitis, liposomes should be preferred to conventional formulations [172]. These conclusions were confirmed by another study, with a ciprofloxacin liposomal system. The authors demonstrated that the liposomes improved the intraocular bioavailability of the drug. MLV showed a concentration of drug in vitreous higher than the MIC90 value after three days of the intravitreal injection, and after 14 days, they found no drug in the vitreous [173].

In a recent study, minocycline-liposomes were developed for a subconjunctival injection and compared to free minocycline injection. They obtained SUV (Small Unilamellar Vesicles) with an average particle size of 80 nm ± 20 nm. The authors concluded on a higher release of drug than free minocycline in the retina with loaded liposomes [174].

##### Niosomes for Antibiotic Delivery

Antiglaucoma therapy requires a continuous and chronic administration of antibiotics. To improve the low corneal penetration and bioavailability of drugs in conventional ocular forms, azetazolamide-niosomes were tested as ocular drug delivery vesicles. Span^®^ 40 or 60 and CH were used in different molar ratios. The results showed that the ratio 7:6 (Span^®^ 60:CH) made MLV and had the higher entrapment efficiency. The formulation showed a high retention of drug with 75% of active substance in the vesicles after 3 months at +4°C. The intraocular pressure (IOP) was measured to establish the treatment efficacy due to the antimicrobial activity of acetazolamide. There was a better decrease of IOP with the niosomes compared to the free drug solution. The most effective molar ratio was 7:4 (Span^®^ 60:CH) with a prolonged decrease of IOP. In addition a reversible irritation in the rabbit’s eyes was noted with no major change in ocular tissues [175].

Another study explored acetazolamide-niosomes coated with Carbopol^®^ (bioadhesive effect) for a glaucoma treatment. The low solubility (0.7mg/mL) and low permeability coefficient of the drug require frequent administration. They compared the coated niosome with an aqueous suspension with 1% *w/v* of Tween^®^ 80 as dispersing agent. They demonstrated a concentration of acetazolamide in the aqueous humor (determined by a microdialys method) two fold higher with niosomes than using aqueous suspension and a longer effect; 6 h for the niosomes against 3 h for the aqueous suspension [176].

Gentamicin is a water-soluble antibiotic which was studied in a niosomal system with Tween^®^ (60 or 80) or Brij 35, CH and dicetylphosphate. With in vitro drug release, the study demonstrated a higher drug concentration inside the vesicles and slower drug release compared to the aqueous solution. They observed that the size of vesicles depended of amount of cholesterol and surfactant type. The molar ratio of 1:1:0.1 (Tween^®^ 60:CH:dicetylphosphate) had the higher entrapment efficiency (92%) and the higher release rate of drug 8 h after administration (66%) with no sign of ocular irritation [177].

More recently, a study confirmed this conclusion. Ciprofloxacin-niosomes were developed with different concentrations of Span^®^, Tween^®^ and CH to treat conjunctiva and corneal ulcer. They obtained a ranged size from 8.6 to 61.3 μm and an entrapment efficiency of 74% and demonstrated that the MIC values with niosomes were 2-fold higher compared to the free ciprofloxacin. In addition, the authors concluded of the higher release of drug for the combination of Span^®^ and Tween^®^ [178].

## 5. Conclusions

Topical eye drops represent 90% of all ocular dosage forms. In recent years, medical and pharmaceutical researchers have made major advances in the field of ophthalmic administration and in ocular drug delivery systems. New ocular drug delivery systems have great potential to improve drug bioavailability in the eye. Limitations of the ocular barriers are major issues to solve for an optimal formulation. Active substance limitations are decreased with the choice of an adaptable form and composition. Patient compliance improves with a tolerable and non-irritating formulation; this parameter is primary for an acceptable administration.

This review showed various development studies of ocular delivery forms. Many studies explored the possibility to decrease the side effects of ocular barrier to prolong ophthalmic residence of the drugs in the eyes, to improve the bioavailability of the active substances and to enhance ocular penetration. Various antibiotics with different characteristics were tested with different delivery systems in order to improve their ophthalmic bioavailability. Antibiotic administration required optimal antimicrobial efficacy. These drugs are used in eye surgeries, anterior segment and posterior segment diseases. Some improvements to limit the impact of the antibiotic’s disadvantages on the eye are under study and under development. Existing forms and new shapes make it possible to increase the ocular therapy efficacy. In the next few years, drug development allowing local action without the need for systemic passage will decrease the frequency of administration, dosage of the drug and improve patient compliance.

## Figures and Tables

**Table 1:** Examples of thermosensitive, pH-sensitive and ion-sensitive polymers used for ophthalmic hydrogel formulations.

Type	Polymers	References
Thermosensitive gels	Negative: Pluronics, poly(N-isopropyl acrylamide) Positive: poly(acrylic acid), polyacrylamide, Reversible: poloxamer, chitosan, hydroxyl propyl méthyl cellulose	[30,31,32,33]
pH-sensitive gels	Cellulose acetate and derivatives Carbomer Magrogol Pseudolatex Polymethacrylic acid	[29] [34] [35]
Ion-sensitive gels	Alginate sodium gellan gum (Gelrite^®^)	[3] [29]

**Table 2:** Example of colloidal system for ocular drug delivery of antibiotics.

Formulation	Antibiotic	Anterior (AS) or Posterior (PS) Segment	Disease Targeted	References
Microemulsion	Chloramphenicol	AS	Trachoma Keratitis	[138]
	Moxifloxacin	AS	Bacterial keratitis	[139]
Nanoemulsion	Polymixin B	AS	Ophthalmic infection	[142]
Nanoparticles	Tobramycin	AS + PS	Bacterial infection *Pseudomonas aeruginosa*	[163]
	Levofloxacin	AS	Bacterial infection *S. aureus* and *E. coli*	[161]
Liposomes	Ciprofloxacin	PS	Bacterial endophthalmitis	[173]
	Distamycin A	AS	Herpes simplex virus	[165]
Niosomes	Acetazolamide	AS	Glaucoma	[175]
	Ciprofloxacin	AS	Conjunctiva + corneal ulcer	[178]
